# Ethambutol optic neuropathy

**DOI:** 10.3389/fneur.2025.1626909

**Published:** 2025-09-11

**Authors:** Padmaja Sudhakar, Kishor Acharya, Tonse Ashwini Kini

**Affiliations:** ^1^Department of Neurology and Ophthalmology, University of Kentucky, Lexington, KY, United States; ^2^Centennial Neuroscience, Nashville, TN, United States

**Keywords:** optic neuropathy, ethambutol, mycobacteria, arabinosyl transferase, antitubercular therapy

## Abstract

Ethambutol is an antitubercular drug that is commonly used in the areas of the world that is endemic to tuberculosis. It is used in infections caused by *Mycobacterium tuberculosis* and some non-tuberculous infections caused by Mycobacterium species. Ethambutol related toxic optic neuropathy remains one of the most concerning adverse effects of the medication in addition to other non-specific side effects like peripheral neuropathy, nausea, vomiting, joint pain and rash. Recently the guidelines for treatment of tuberculosis in some countries where tuberculosis (TB) is endemic have been revised to allow for fixed dose combinations (wherein a single tablet contains a fixed strength of isoniazid, rifampicin, pyrazinamide, and ethambutol) and longer duration of treatment with ethambutol. Thus, it is anticipated that there will be an increase in ethambutol toxic optic neuropathy. In this minireview we plan to discuss the clinical features, neuro-ophthalmic evaluation, screening and diagnosis of ethambutol toxic optic neuropathy. We will also discuss the available treatment options for this condition.

## Introduction

Ethambutol is a well-known potent bacteriostatic anti-tubercular agent that has been effectively used for several decades to treat tuberculosis as well as non-tubercular Mycobacterial infections. It is a metal chelator and disrupts cell wall synthesis in mycobacteria by inhibiting arabinosyl transferase.

Involvement of optic nerve was noted early on with use of Ethambutol in 1961. Drug related side effect profile is cumulative and directly correlates to dose and duration of use (1–4)^.^ Time of onset is variable and can occur anytime during the treatment course, but on average could range at 3–5 months from the time of drug initiation (5–8). WHO recommends an initiation dose of 15 to 20 mg/kg/day. Nontuberculous mycobacterial infections like *M. avium* complex and *Mycobacterium kansasii* are also treated with ethambutol and have different dosing (9). The American Thoracic Society recommends a range of 15 mg/kg per day for *M. kansasii* to 25 mg/kg per day for macrolide-resistant *M. avium* complex (9).

Ethambutol was initially recommended for 2 months in the intensive treatment phase of tuberculosis (TB). But with the increase in drug resistance, the World health organization (WHO) in 2009 recommended use of ethambutol in the maintenance phase. The revised national tuberculosis control program (RNTCP) introduced this change in 2016 in India and increased the duration of the ethambutol intake from 2 months to 6 months. Dosing was changed from three times a week to daily for 6 months (10, 11).

The average incidence of Ethambutol optic neuropathy (EON) is 1% at WHO recommended daily dose (12), and the risk substantially increases with higher doses such as 30 mg/kg/day or with any increase in dose, and in the presence of comorbidities like renal dysfunction which can reduce drug excretion and raise serum levels, smoking, poorly controlled diabetes, hypertension, age above 65 years, malnutrition, and concomitant use of isoniazid (13, 14). Sadun and Wang (15) remarked that adverse effects of the drug can occur if changes in body weight and a decline in renal function during the treatment course are not taken into account. Patients being treated for non-tubercular infections may be at a higher risk of EON due to receiving higher doses.

The estimated incidence of ethambutol-related ocular toxicity, according to some studies ranges from 1–2.5% for dosage of 15 mg/kg per day, 5–6% for dosage of 25 mg/kg/day and 18% for dosage of 35 mg/kg/day.

Studies have shown that the new Extended treatment protocol regimen for Tuberculosis (TB) has resulted in an overall increased incidence of EON in endemic countries (13–16). Authors of a large 10 year study from South Taiwan suggest that the true incidence of EON could be higher since ophthalmologic examinations were only available for approximately one-fourth of patients (14, 17).

## Mechanism of injury

The exact mechanism of ethambutol induced optic neuropathy remains unknown. Ethambutol inhibits arabinosyl transferase, an important enzyme in mycobacterial cell wall synthesis. Ethambutol and its metabolite 2,2′-ethylenediamino-dibutyric acid (EDBA) are both effective metal chelators. Human mitochondrial DNA is similar to that of mycobacteria. Ethambutol may be chelating the copper ion of the enzyme cytochrome-c oxidase in mitochondria of optic nerve axons and thus disrupt oxidative phosphorylation in human mitochondria. Ethambutol may also inhibit lysosomal activation due to the chelation and accumulation of zinc (18–20). The metabolite of ethambutol 2,2′-ethylenediamino-dibutyric acid (EDBA) is known to have reduced ocular clearance and thus high local concentrations as compared to ethambutol itself. Both ethambutol and its metabolite by their chelating properties are hypothesized to cause the optic neuropathy (21)^.^

## Clinical presentation and ophthalmic exam findings

**Figure 1:**
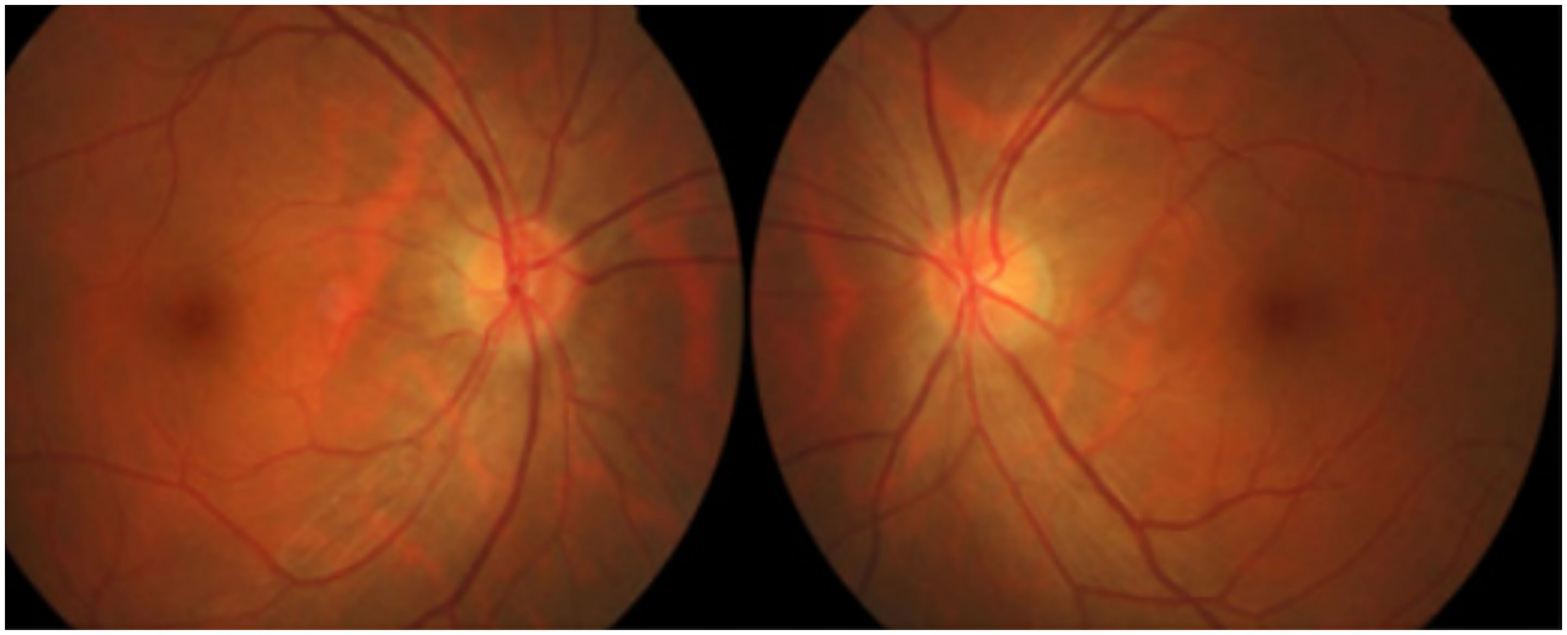
Optic disk images showing hyperemia in a patient with ethambutol optic neuropathy. Courtesy: Zhang et al. (44).

**Figure 2:**
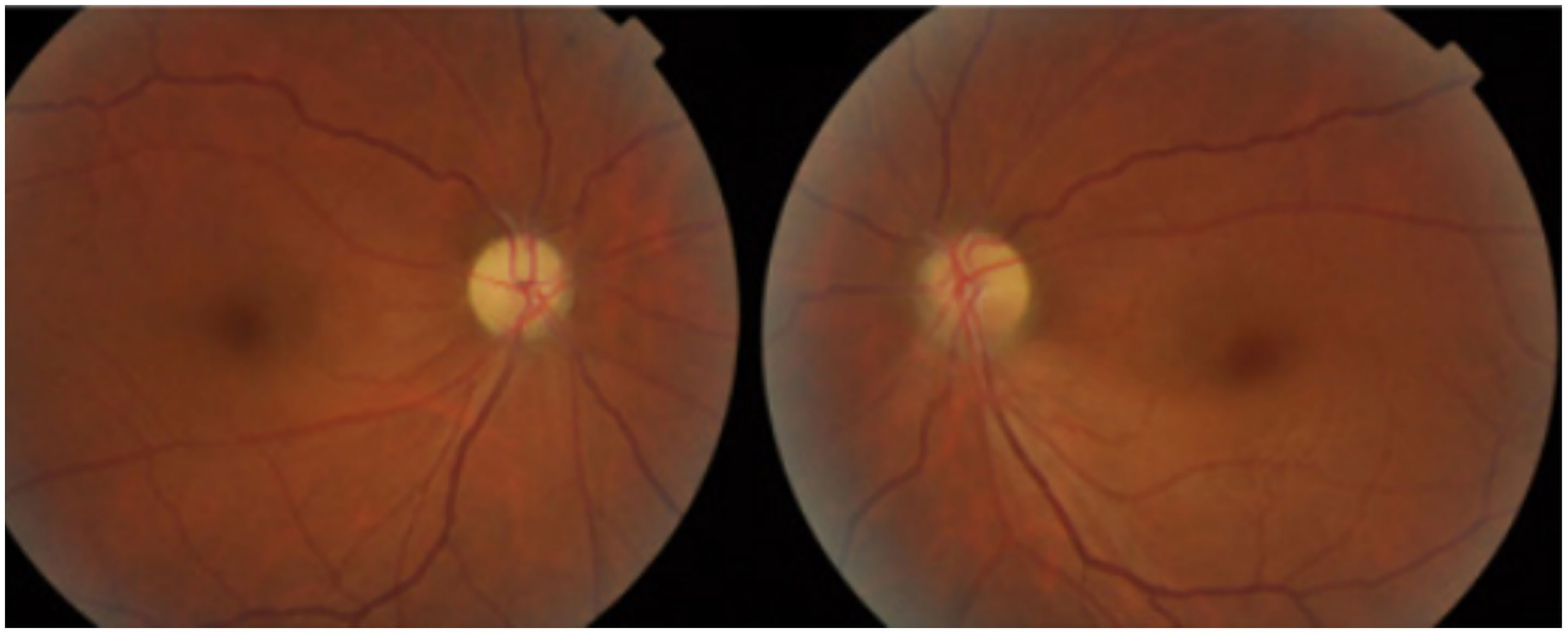
Optic disk images showing pallor in a patient with ethambutol optic neuropathy. Courtesy: Zhang et al. (44).

Patients present with bilateral, subacute, painless, progressive loss of central vision. In addition to blurred vision, they may report difficulty distinguishing colors, or report frequent changes in spectacles or contact lens prescription (7). Examination may reveal that the visual acuity is compromised and color vision testing could reveal dyschromatopsia. Patients may demonstrate difficulty in distinguishing red and green colors but may also have trouble with blue and yellow colors (6, 7, 22, 23). Due to bilateral symmetric involvement, pupil exam often does not reveal a relative afferent pupil defect, however in case of sequential involvement, relative afferent pupillary defect might be seen. Fundus exam tends to be normal during the initial stage of the disease but can become abnormal as disease progresses and optic disk pallor may develop. Presence of optic disk pallor at presentation indicates poor prognosis. Fundus examination can show hyperemia around the disk (Figure 1), pallor (Figure 2) and peripapillary hemorrhage in the later stage of the disease.

**Figure 3:**
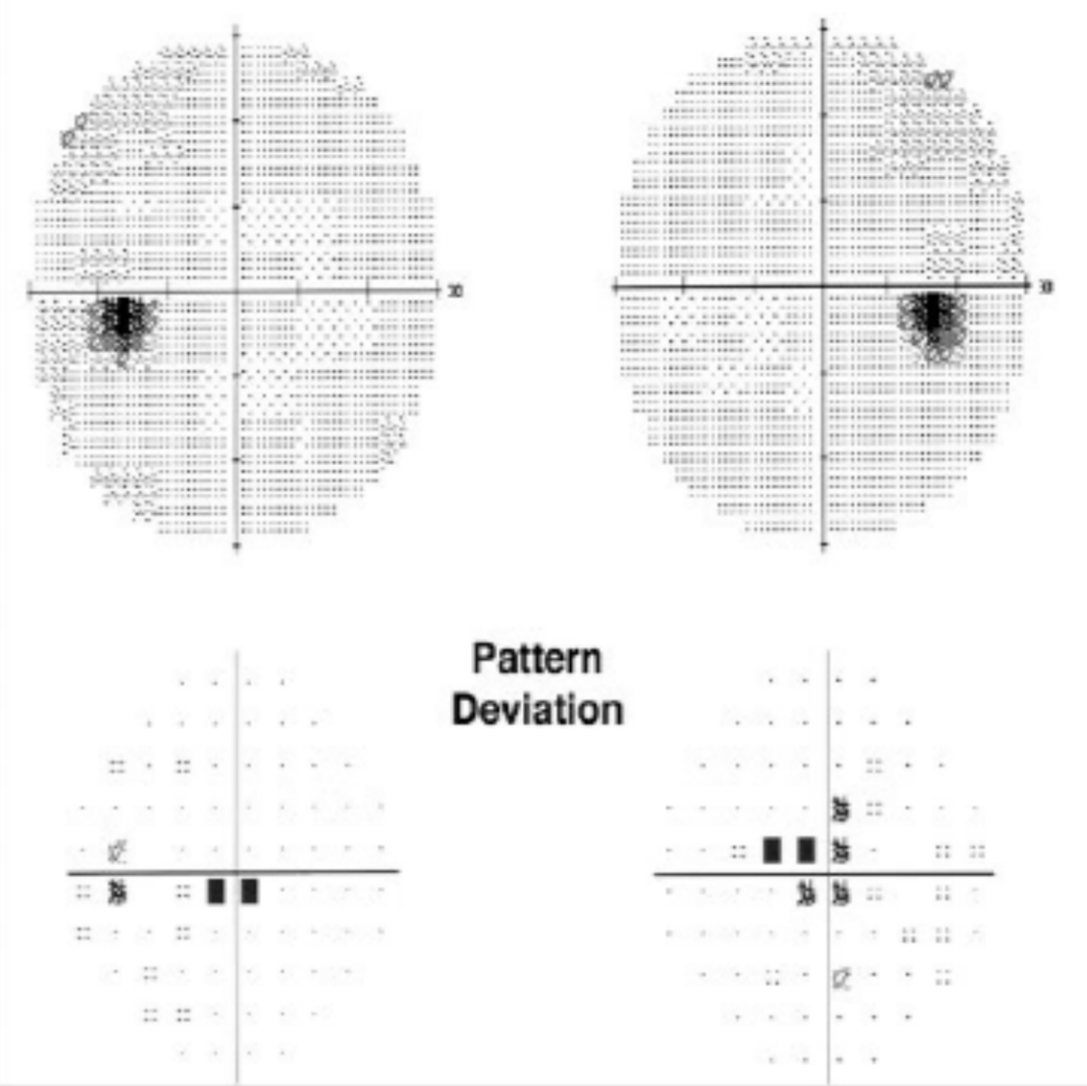
Humphrey visual fields showing bilateral central scotoma in a patient with ethambutol optic neuropathy. Courtesy: Kho et al. (25).

**Figure 4:**
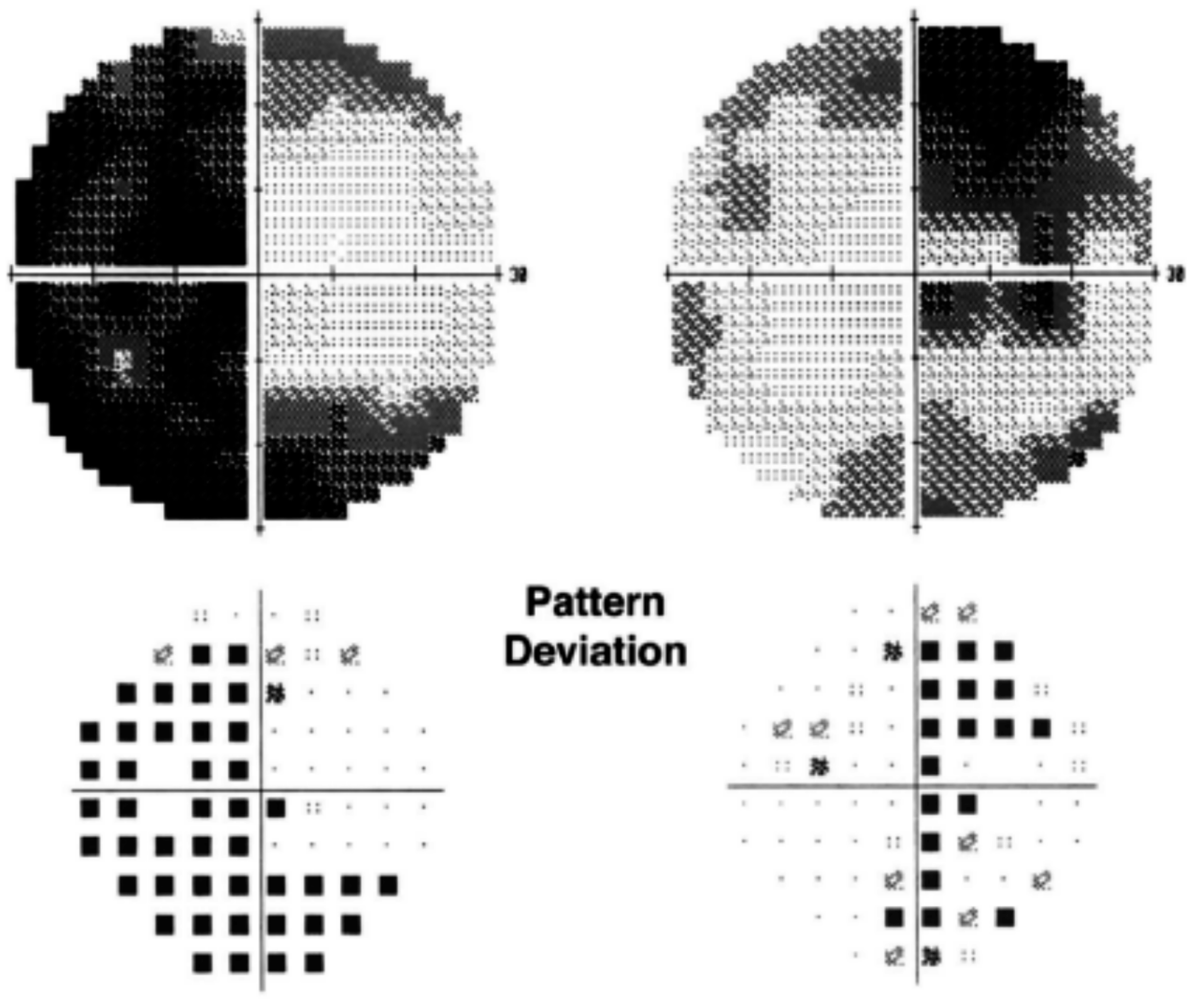
Humphrey visual fields showing bitemporal hemianopia in a patient with ethambutol optic neuropathy. Courtesy: Kho et al. (25).

Automated perimetry reveals central or centrocecal scotomas (Figure 3) and less commonly peripheral visual field defects as well. Bitemporal field loss with involvement of optic chiasm has also been demonstrated (24–26) (Figure 4).

**Figure 5:**
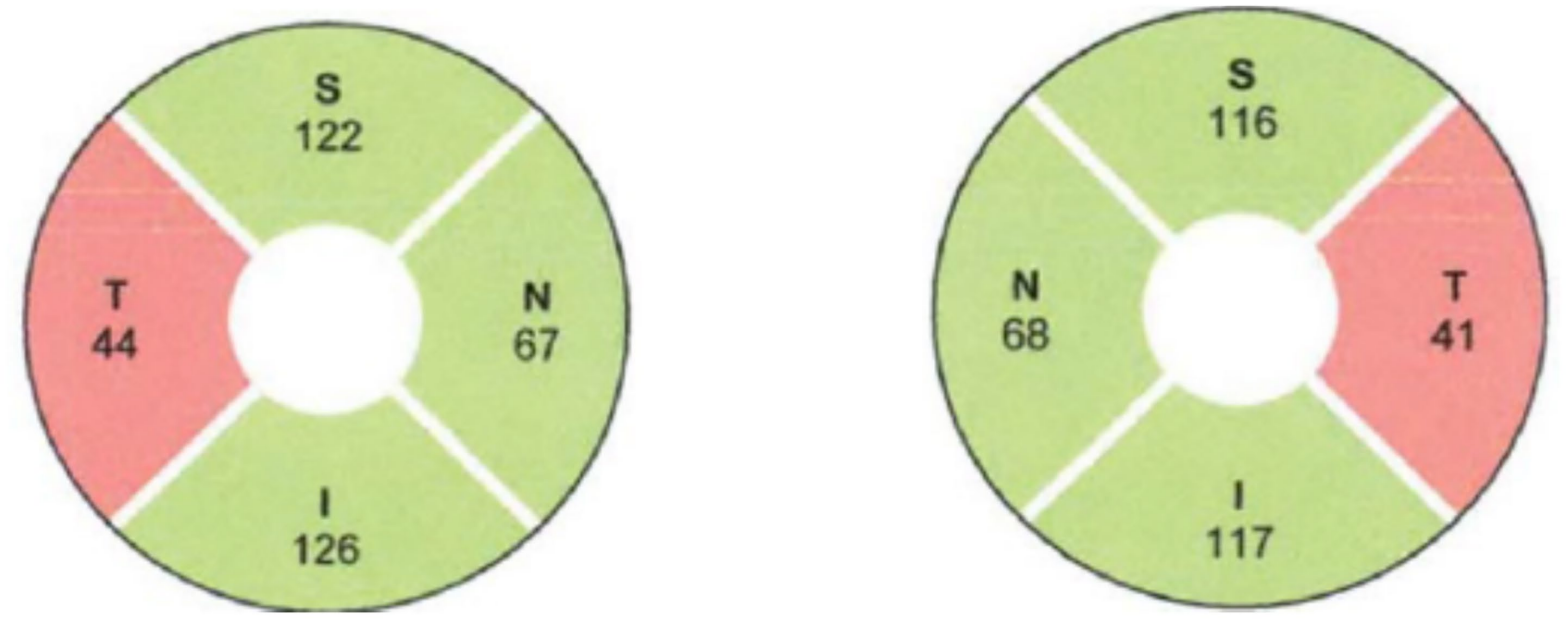
Retinal nerve fiber layer analysis showing bilateral temporal retinal nerve fiber layer thinning in a patient with ethambutol optic neuropathy. Courtesy: Bouffard et al. (45).

Optical coherence tomography (OCT) analysis could help identify early EON changes that could be missed on funduscopic examination. Retinal nerve fiber layer (RNFL) and ganglion cell inner plexiform layer (GCIPL) thinning may be seen in upto 20–79% with a predilection for temporal quadrant (Figure 5) followed by superior quadrant (27–29). This appears to be consistent with prior histopathologic studies wherein toxic/nutritional optic neuropathies have a tendency to affect the papillomacular bundle earlier involving the parvo-cellular axons due to high energy demand from their mitochondria which are targeted in toxic optic neuropathies (30).

It is important to know that the findings on OCT are not specific for ethambutol optic neuropathy (31–35). Patients with EON may show ganglion cell inner plexiform layer (GCIPL) thinning whereas patients without EON showed no significant changes in GCIPL thickness (34). OCT is useful in early stages of EON, in follow-up of patients with EON and in assessing visual prognosis. RNFL thinning on follow-up as early as 2 months of treatment could be a sign of progressive damage (36). The utility of OCT in reliably screening for subclinical EON needs to be further studied.

Pattern reversal Visual evoked response (VER) can show an increase in P100 latency or amplitude in patients with EON (7, 33). A study showed that 34.8% of patients taking ethambutol had increased latency beyond 107 ms, 2 SD beyond the control mean of 97 ms (37).

While visual fields, OCT, and Pattern reversal VER can help identify risk of toxicity early, their role in detecting subclinical disease still remains unclear.

Multifocal electro-retinogram can be used to rule out a retinal pathology and may be able to detect retinal damage secondary to ethambutol. Magnetic resonance imaging of head and orbit may be performed to rule out any compressive or demyelinating pathology, however in EON itself, it may be normal. Some patients have been noted to have enhancement of optic nerve at the chiasm or have T2 FLAIR hyperintensity in optic chiasm or optic tract (38, 39).

## Ethambutol optic neuropathy in children

Interestingly, the prevalence of EON is much less in children than in adults. Previously ethambutol was seldom used in children, especially under age of 5 years. But in 2010 WHO recommended a dose of 20 mg/kg/day instead of 14 mg/kg/day in children (40). But serum concentration of ethambutol is lower in children than adults for a given dose (41). Therefore, EON in children is very rare. However, ophthalmology screening for EON should still be carried out in children receiving ethambutol.

## Management

There is unfortunately no proven treatment for EON. Screening with early detection of EON and stopping the offending drug is the mainstay of the treatment plan.

The authors agree with the consensus guidelines provided by Saxena et al. (11) who state there is a need to highlight the potential risk of optic neuropathy from the use of ethambutol, particularly in view of the longer periods of use in endemic countries per guidelines from revised national tuberculosis control program RNTCP and WHO.

Toxic optic neuropathy may occur at any time with ethambutol, but most often, it is seen after 3–5 months of use. Patients receiving less than 15 mg/kg per day of ethambutol are much less likely to develop EON. But it has been reported to occur in patients receiving lower doses. All patients taking ethambutol of any dose should undergo regular eye exams for screening for EON. Comprehensive eye exams should test visual acuity, visual fields, color vision, contrast sensitivity, perform dilated fundus exam and OCT and VEP if available.

Chances of recovery are higher when EON is identified early and necessary measures such as dose reduction or switching to alternate therapy are taken to halt any further cumulative effects from the medication. Early diagnosis is critical to prevent severe vision loss, and awareness is necessary among screening Ophthalmologists and Optometrists to identify the signs of EON. The tests that may help detect ethambutol toxicity at the subclinical stage include a change in average RNFL thickness, most pronounced in the temporal quadrant (initial increase followed by decrease) and GCIPL thinning on OCT, reduction in visual field index, presence of nerve fiber bundle pattern of visual field defect, and prolonged VEP latency.

If ethambutol is stopped early, visual recovery may occur in at least one third of patients. Studies have shown that there may be improvement in two or more lines on Snellen’s chart (7, 42, 43) and 30 to 64% can have improvement in vision over several months (7, 8, 22). Irreversible vision loss can occur despite close monitoring and prompt discontinuation of the drug (43). Chai and Foroozan (27) reported a continued decline in RNFL thickness even following cessation of Ethambutol. Increased age may adversely affect the chances of recovery from EON (8). Small case series showed that the recovery rate of EON was only 20% in those over age 60, and 80% for those under age 60.

If EON is suspected, the ophthalmologist should immediately contact the ethambutol prescribing physician to stop the agent and choose an alternative such as fluoroquinolones. Drug-resistant TB is an increasing concern, being met, in part, with repurposed drugs (like moxifloxacin, levofloxacin, linezolid, clofazimine, and beta-lactams) and new drugs (like bedaquiline, pretomanid, and delamanid). Use of such drugs could be considered in patients where ethambutol toxic optic neuropathy is identified. Adverse effects such as QTc interval prolongation and drug penetration into organism sanctuaries, such as the central nervous system, bone, and pulmonary TB cavities remain important challenges to be met.

## Prevention

It is important to educate primary care providers and all ethambutol prescribing physicians and patients about the potential ocular toxic side effects. A baseline ophthalmologic exam prior to drug initiation would be ideal. Periodic eye exams (even on a monthly or three monthly basis) while being treated with ethambutol should be encouraged. In endemic countries where EON is common, ophthalmologists could consider screening all patients for EON and doing a periodic comprehensive eye exam to include visual acuity, visual fields, color vision, contrast sensitivity, dilated fundus exam and OCT and VEP if available. Patients could use an Amsler grid or a pocket Snellen chart to monitor their vision at home while on ethambutol and get an immediate eye exam if they notice subtle changes in their vision.

## Conclusion

Ethambutol is one of the most common and well known drug induced toxic optic neuropathy. It causes irreversible blindness which may be preventable with early recognition and intervention. VEP and OCT can be useful tools in detecting clinical disease but utility in detecting subclinical disease and their role in prevention is not well established. Early detection of optic neuropathy and withdrawal of the drug remains the mainstay of management at this time. Educating prescribing physicians and ophthalmologists on early signs and symptoms of EON is important in early detection and prevention of vision loss.
